# Investigating the Roles of Coat Protein and Triple Gene Block Proteins of Potato Mop-Top Virus Using a Heterologous Expression System

**DOI:** 10.3390/ijms25136990

**Published:** 2024-06-26

**Authors:** Hira Kamal, Kasi Viswanath Kotapati, Kiwamu Tanaka, Hanu R. Pappu

**Affiliations:** Department of Plant Pathology, Washington State University, Pullman, WA 99163, USA; hira.kamal@wsu.edu (H.K.); viswanathkotapati@gmail.com (K.V.K.); kiwamu.tanaka@wsu.edu (K.T.)

**Keywords:** tuber necrosis, viral transmission, symptom development, potato mop-top virus, potato virus X, hypersensitive response

## Abstract

Potato mop-top virus (PMTV) is an emerging viral pathogen that causes tuber necrosis in potatoes. PMTV is composed of three single-stranded RNA segments: RNA1 encodes RNA-dependent RNA polymerase, RNA2 contains the coat protein (CP), and RNA3 harbors a triple gene block (TGB 1, TGB2, and TGB3). CP plays a role in viral transmission, while TGB is known to facilitate cell-to-cell and long-distance systemic movement. The role of CP in symptom development, specifically in the presence of TGB genes, was investigated using potato virus X (PVX) as a delivery vehicle to express PMTV genes in the model plant *Nicotiana benthamiana*. Plants expressing individual genes showed mild symptoms that included leaf curling and crumpling. Interestingly, symptom severity varied among plants infected with three different combinations: CP with TGB1, CP with TGB2, and CP with TGB3. Notably, the combination of CP and TGB3 induced a hypersensitive response, accompanied by stunted growth and downward curling and crumpling. These results suggest the potential role of TGB co-expressed with CP in symptom development during PMTV infection. Additionally, this study demonstrates the use of the PVX-based expression system as a valuable platform for assessing the role of unknown genes in viral pathogenicity.

## 1. Introduction

Potato (*Solanum tuberosum*) is one of the world’s most essential staple foods in our daily diet [1]. However, this vital crop faces an ongoing threat from numerous viral and fungal pathogens, which pose a constant challenge to the potato industry, resulting in reduced yield and compromised quality [2,3,4]. Among these threats is the potato mop-top virus (PMTV), a member of the *Pomovirus* genus in the *Virgaviridae* family. PMTV induces tuber necrosis with the appearance of arcs and streaks, commonly referred to as “spraing” [5,6]. PMTV spreads through a Plasmodiophorid protist, *Spongospora subterranea* f. sp. *subterranea,* abbreviated as “*Sss*”, the causal agent of powdery scab disease [7,8]. Notably, PMTV can persist in the resting spores of *Sss* for extended periods, making its elimination a challenging endeavor [9]. 

Potato tuber infection caused by *Sss* and PMTV is becoming more common in the United States and could become a threat for potato production. The infection level in the field could be tested using real-time quantitative reverse-transcription PCR (qRT-PCR) to identify susceptible cultivars [10]. PMTV can be transmitted from infected tubers to daughter plants with variations in transmission rate that face several obstacles and challenges to control this disease [11]. Among effective management strategies, planting PMTV-free seed potato is the most preferable option after testing the seeds and the soil with certain specific tests such as RT-PCR and ELISA [10,12]. Crop rotation in five- or multiple-year intervals between potato crops and non-potato crops may also reduce the spread of PMTV present in the soil. In addition, timely reporting of this virus to PMTV technical advisory groups could instigate biosafety responses to invade PMTV spread [13]. 

The genome of PMTV consists of three RNA segments: RNA-1 (6.043 kb), encoding RNA-dependent RNA polymerase (RdRp); RNA-2 (3.134 kb), containing the coat protein (CP); and RNA-3 (~2.964 kb), housing the triple gene block, TGB1, TGB2, and TGB3 [14,15,16]. RdRp plays a crucial role in replication, while CP and the larger CP-readthrough (RT) protein in RNA-2 are necessary for vector transmission [15]. RNA-3 comprises four open reading frames (ORFs), with TGB1 to 3 involved in cell-to-cell and systemic movement of the virus. The fourth ORF encodes an 8K putative cysteine-rich protein that enhances the virulence and suppresses host RNA silencing [17,18]. The genetic diversity of PMTV has been reported from different parts of the world and shows a high sequence identity among isolates from Asia, Europe, and North America [19,20,21,22,23,24,25]. These data have used RdRP, TGBs, and CP sequences to investigate PMTV’s genetic diversity and distribution based on selection pressure [26]. Numerous studies have been conducted to determine the function of each PMTV gene. The interaction among TGB proteins (TGB1, TGB2 and TGB3) facilitates cell-to-cell and long-distance movement for successful viral infection [26,27,28,29]. Using a PVX-based expression vector [30], the significance of the N-terminal domain of TGB1 in nucleolar localization and long-distance movement during PMTV infection was determined. Meanwhile, CP in RNA-2 was considered for vector-based virus transmission into the plant tissue [5]. 

Considering the effect of viral infection above the ground, phenotypic data of different PMTV isolates have been grouped into mild, medium, and severe, based on the symptoms they cause [31]. Mild symptoms may include leaf deformation and shortened internodes, while disease symptoms produce necrosis inside tuber flesh [32,33]. It was hypothesized that genes present in RNA-2 and RNA-3 assist each other to induce foliar symptoms. Here, in order to determine the role of specific genes or sets of genes for symptom development, a PVX-based expression system was used to express TGB1, TGB2, TGB3, and CP individually and in various combinations in *N. benthamiana,* and we evaluated the plant’s response. Data collected in this study showed that plants expressing TGB1 and TGB2 in conjunction with CP exhibited mild symptoms, whereas the presence of TGB3 with CP resulted in severe symptoms in younger uninoculated (systemic) leaves. The findings, based on both phenotypic and molecular data, suggest that CP and TGB, particularly TGB3, exacerbate PMTV symptoms by facilitating viral long-distance movement.

## 2. Results

### 2.1. Effect of Expression of Individual TGBs and CP on Host Response

**Figure 1:**
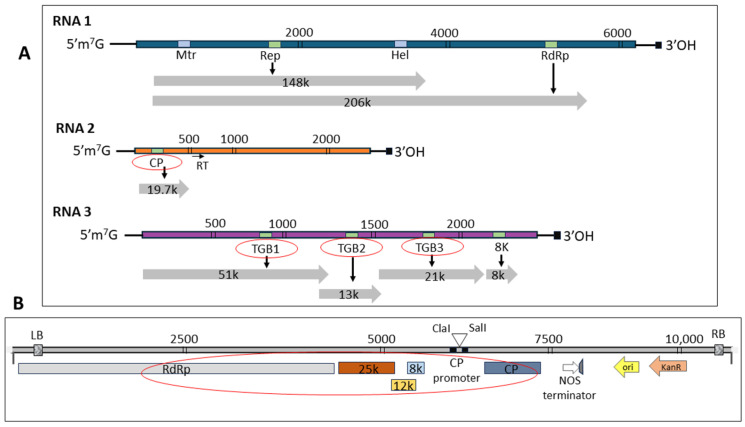
Genome structure of potato mop-top virus (PMTV) and vector map of the potato virus X (PVX)-based expression system. (**A**) Schematic diagram shows PMTV genome map that comprises three RNA segments: RNA-1 (6.043 kb), RNA-2 (3.134 kb), and RNA-3 (~2.964 kb). (**B**) Map of pGR106 vector illustrating the incorporation of the PVX genome (red circle) including genes for RdRp, TGB1 (25 kDa), TGB2 (12 kDa), TGB3 (8 kDa), and CP. The PVX expression cassette is driven by the 35S CaMV promoter, and the PMTV genes were cloned downstream of PVX TGBs and upstream of PVX CP.

In order to express PMTV genes (CP derived from RNA2 and TGBs from RNA3, as illustrated in Figure 1A), the pgR106 vector was utilized as the PVX-based expression system (Figure 1B). The expression cassette within the PVX expression vector is driven by the 35S CaMV promoter and encompasses PVX RdRP, along with three movement proteins (25 kDa, 12 kDa, and 8 kDa), as well as CP. The PMTV genes were separately cloned between the PVX movement proteins and the PVX CP. 

**Figure 2:**
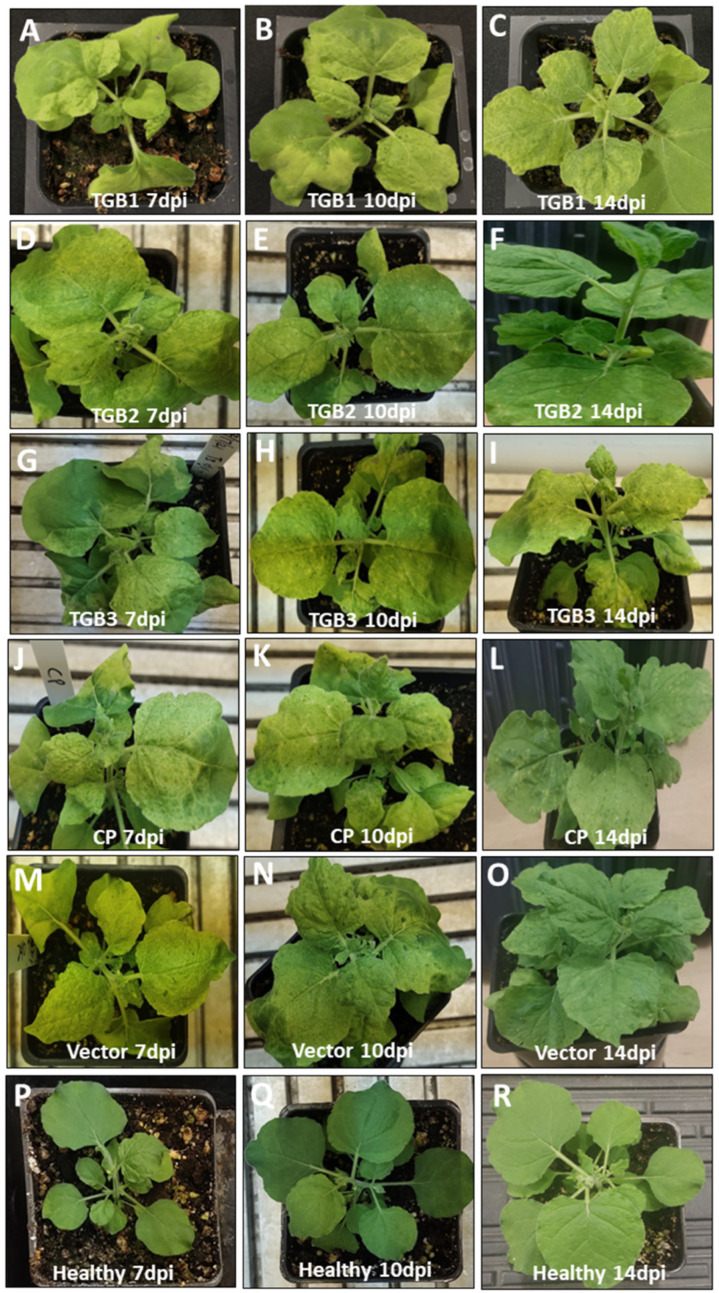
Symptoms induced by potato mop-top virus (PMTV) coat protein and triple gene block encoding genes following infiltration of *Nicotiana benthamiana* plants. The PMTV genes were expressed using a potato virus X-based expression vector. Younger leaves were collected at three different time points, 7, 10, and 14 dpi. (**A**–**C**) Plants inoculated with TGB1 exhibited mild symptoms of curling and crumpling. (**D**–**F**) TGB2 also produced curling and mottling of leaves at all three time points. (**G**–**I**) TGB3-infected plants showed mild to severe symptoms of curling and crumpling of the leaves. (**J**–**L**) Coat protein-infiltrated plants produced mild to severe symptoms. (**M**–**O**) Vector-only plants displayed leaf mottling followed by light green chlorosis and necrotic spots. (**P**–**R**) Healthy plants.

Using a PVX-based expression system, the effect of individually expressing PMTV CP and TGBs on viral symptoms was investigated in *N. benthamiana* plants. At 7 dpi, plants expressing TGB1 started exhibiting mild symptoms including upward and downward leaf curling and crumpling on both inoculated as well as younger, uninoculated adjacent leaves. These symptoms persisted until 10 dpi, gradually diminishing thereafter by 14 dpi (Figure 2A–C). Similar symptoms were observed in plants expressing TGB2, characterized by mild leaf curling with crumpling (Figure 2D–F). Interestingly, plants expressing TGB3 displayed more severe symptoms, including pronounced leaf curling, severe crumpling, and marginal patches on the leaf surface from 7 dpi to 10 dpi (Figure 2G,H). These plants also showed patches of dead cells forming necrotic lesions, a potential manifestation of hypersensitive response (HR) throughout different leaf areas, which gradually subsided by 14 dpi (Figure 2I). Leaves infiltrated with CP alone also produced symptoms ranging from mild to severe at 7 dpi, featuring distinct curling in the local and adjacent younger leaves (Figure 2J). Symptom severity increased between 10 and 14 dpi. Necrotic lesions were observed early from 7 to 14 dpi, although plants eventually recovered from the infection (Figure 2K,L). Plants infected solely with the PVX, vector induced mild mosaic symptoms with light green necrotic spots on the leaves (Figure 2M–O). Mock-inoculated plants did not show any symptoms throughout the observed time points (Figure 2P–R).

### 2.2. Combined Effect of Expression of CP with Each TGB ORF on Infiltrated Plants

**Figure 3:**
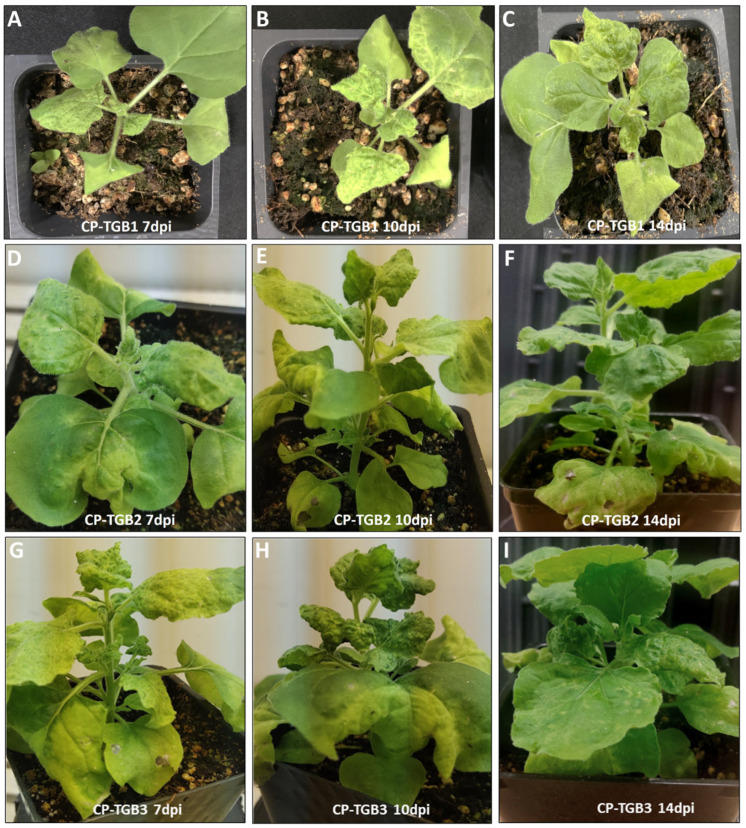
The combined effect of coat protein and triple gene block encoded genes of potato mop-top virus in Nicotiana benthamiana plants. (**A**–**C**) CP-TGB1 plants induced mild symptoms. These symptoms became less obvious at 14 dpi. (**D**–**F**) CP-TGB2 showed severe symptoms especially in younger leaves as compared to the CP-TGB1 plants. (**G**–**I**) Combination of CP-TGB3 induced strong curling and crumpling along with necrosis from 7 dpi to 10 dpi.

CP was co-expressed with each of the three TGB ORFs individually to investigate the impact of CP in conjunction with each TGB ORF on host response. The combinations of CP co-expressed individually with TGB1, TGB2, and TGB3 were labeled as CP-TGB1, CP-TGB2, and CP-TGB3, respectively. Interestingly, plants expressing CP-TGB1 exhibited milder symptoms compared to those infiltrated by TGB1 alone (Figure 3A–C). In the case of CP-TGB2-infiltrated plants, symptoms ranged from mild to severe curling and crumpling across all three time points (Figure 3D–F), whereas CP-TGB3 symptoms were relatively more severe, characterized by leaf curling and crumpling in both locally inoculated and younger, uninoculated leaves (Figure 3G–I). Furthermore, this host response appeared earlier, from day 10 in plants expressing either CP-TGB1 or CP-TGB2, as well as those expressing individual genes.

### 2.3. Transcript Levels of CP and TGB Genes in Inoculated Plants

**Figure 4:**
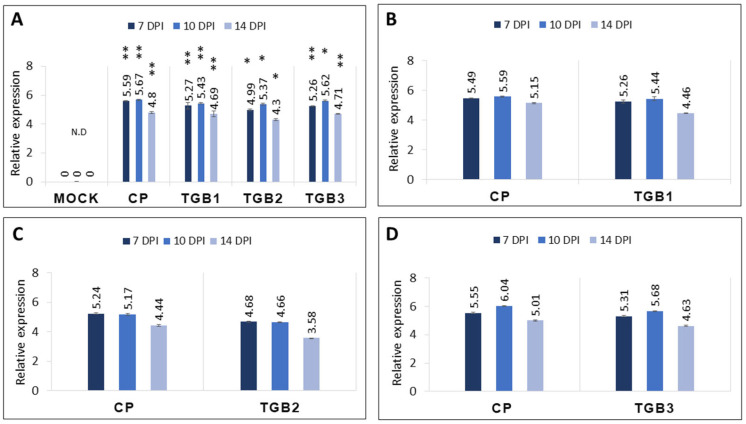
Expression analysis of potato mop-top virus (PMTV) genes in *Nicotiana benthamiana* plants. (**A**) Relative level of transcripts of PMTV-encoded TGB1, TGB2, TGB3 and CP were quantified using qRT-PCR. All genes were expressed at 7 dpi, 10 dpi and 14 dpi. The histogram shows the relative gene expression levels with mean +/− SE after normalization to the reference genes EF1-alpha and phosphatase 2A (PP2A). Asterisks indicate statistically significant differences (* *p* < 0.05; ** *p* < 0.01) compared to the data obtained from the healthy plant. (**B**) Gene expression of PMTV CP and TGB1 was determined in plants infiltrated with CP and TGB1 together. (**C**) CP and TGB2 gene expression from CP-TGB2-infiltrated plants. (**D**) CP and TGB3 gene expression from CP-TGB3-infiltrated plants.

To assess the expression levels of CP and the three TGB ORFs, qRT-PCR was performed on total RNAs extracted from leaves inoculated with the PVX expression vector carrying individual genes (CP, TGB1, TGB2, and TGB3) as well as combinations (CP-TGB1, CP-TGB2, and CP-TGB3). The results revealed minor differences in the expression levels among the genes, with CP consistently exhibiting relatively higher expression compared to TGB1, TGB2, and TGB3 across all three time points (Figure 4A). Additionally, plants co-inoculated with CP-TGB1 displayed slightly elevated levels for both CP and TGB1 compared to those co-infiltrated with CP and TGB2 together (CP-TGB2) (Figure 4B,C). Notably, an upregulation of both CP and TGB3 transcripts was observed at 10 dpi in plants co-inoculated with CP-TGB3 (Figure 4D). Overall, gene expression levels at 14 dpi were consistently lower regardless of the individual or combinations of the PMTV genes.

### 2.4. Serological Testing of CP in Infected Plant Tissue

**Figure 5:**
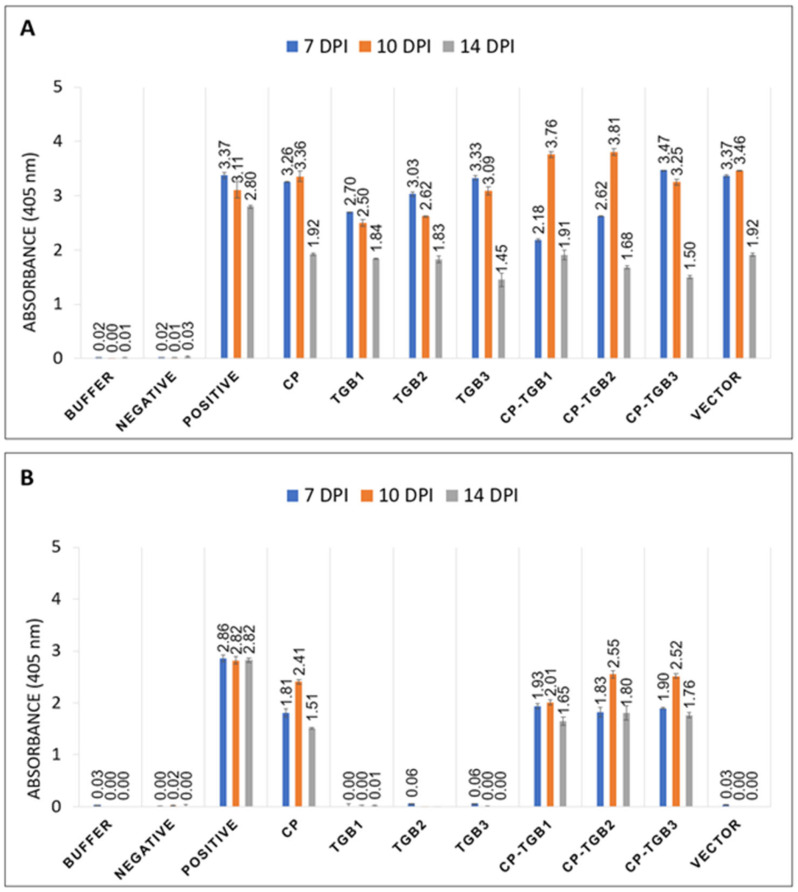
Detection of potato virus X (PVX) (**A**) and potato mop-top virus (PMTV) (**B**) coat protein using PVX- and PMTV-specific antisera by ELISA. (**A**) OD-405 showed PVX-CP was detected among all infected samples and empty vector. (**B**) PMTV-CP antisera detected CP in plants infiltrated with constructs CP-, CP-TGB1-, CP-TGB2-, and CP-TGB3-infected leaves. Positive and negative represent extracts from known infected and virus-free samples, respectively.

ELISA was used to determine the relative levels of PVX and PMTV. In the case of PVX CP, similar absorbance readings were obtained using PVX-specific polyclonal antiserum for both empty vector and leaf samples infiltrated with PMTV TGB1, TGB2, TGB3, and CP (Figure 5A). These absorbance levels remained consistent at 7 dpi and 10 dpi, while a lower absorbance value was obtained at 14 dpi. In the case of PMTV CP ELISA, plants infiltrated with CP-TGB1, CP-TGB2, and CP-TGB3 exhibited similar absorbance values to those with PMTV CP alone (Figure 5B).

### 2.5. Sequence Comparison of PVX and PMTV Genes

**Figure 6:**
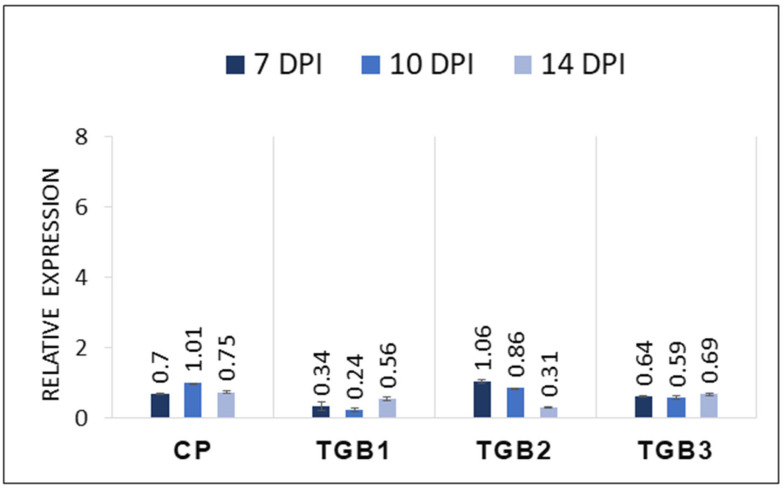
Attestation of experimental controls for data validation. Potato virus X empty vector was tested with PMTV gene-specific primers to cross-reference the phenotypic data. qRT-PCR data showed negligible expression level for PMTV-CP and TGB encoded genes in PVX vector-only infiltrated plants.

PMTV genes were expressed using a PVX backbone, where PVX encodes 8 kDa, 12 kDa, 25 kDa, and CP, while PMTV genome codes for TGB1 (51 kDa), TGB2 (12 kDa), TGB3 (25 kDa), and CP (Figure 1A,B). Therefore, it was necessary to ascertain whether the symptoms observed in infiltrated leaves were solely from the PMTV-encoded genes. This validation was necessary despite the minimal symptoms induced by PVX alone (vector-only control in Figure 2M–O). Identifying regions of similarity at the protein level for PMTV with PVX genes was carried out using a pairwise sequence alignment tool [34,35]. The results indicated relatively low identity. For example, PMTV TGB1 exhibited 0.5% and 14% identity with PVX 12 kDa and 25 kDa proteins, respectively. Similarly, PMTV TGB2 displayed an identity of 23% and 10% with PVX 12 kDa and 25 kDa, respectively. PMTV TGB3 showed only 17% and 18% identity with PVX 12 kDa and 25 kDa, respectively. However, in the case of CP, both PMTV CP and PVX CP showed 38% identity at the protein level. Furthermore, primers specific to PMTV genes (used in Figure 4) were used in qRT-PCR to evaluate the possibility of cross-detection of PMTV genes in leaves infiltrated with the PVX empty vector. The high Ct value (>38) obtained from the infected leaves at 7 dpi, 10 dpi, and 14 dpi confirmed the low expression of PMTV genes (Figure 6), suggesting that the symptoms observed in infected plants could be primarily attributed to PMTV CP and TGB genes, as validated by qRT-PCR (Figure 4). 

## 3. Discussion

Genome sequence information within PMTV isolates revealed two distinguishable variants of RNA-CP found in different combinations and mixed infections [6]. This past study further focused on different sequence variations among RNA-CP and RNA-TGB, which were associated with symptom-based and symptomless infection. Data collected from the field showed that the combination of RNA-CP and RNA-TGB is responsible for different PMTV strains infecting potato tubers with spraing symptoms [21]. However, no data are available to determine if these two PMTV RNA segments are also involved in foliar symptoms. 

In the present study, it was hypothesized that PMTV-induced foliar symptoms are the result of interaction among RNA-2 and RNA-3 encoded genes. To determine the specific genes involved in symptom induction during infection, a PVX-based expression system was used [36]. This heterologous system facilitated the investigation of the individual and combined effects of PMTV genes, with a focus on elucidating the role of RNA-2-encoded CP in conjunction with RNA-3-encoded TGB genes during long-distance movement of the virus. 

Previous reports have indicated that foliar symptoms from PMTV infection are influenced by environmental factors such as soil moisture, temperature, and humidity [37,38]. Studies have shown that 13–59% of PMTV infections result in foliage symptoms [39]. However, the absence of symptoms does not guarantee the absence of PMTV infection [13]. The objective of this study was to explore the involvement of RNA-2-encoded CP along with RNA-3-encoded TGB genes involved in foliage symptoms during PMTV infection. 

Previous studies have shown that TGB1 aids in the localization of viral RNA to the plasma membrane and plasmodesmata [26,27,40]. Moreover, the interaction between TGB2 and TGB3 facilitates viral movement to the cell periphery and plasmodesmata through the endoplasmic reticulum network [28,29]. Previous findings suggest that viral cell-to-cell trafficking relies on the N-terminal domain of TGB1 and two transmembrane domains in TGB3, while CP has no involvement in systemic movement [40,41,42,43]. However, the involvement of CP-RT in virus transmission by the soil-borne vector complicates the understanding of this mechanism. Earlier research indicates two possible mechanisms for the movement of PMTV RNA segments: (1) by forming a ribonucleoprotein (RNP) complex with TGB1, in the absence of CP, to transport all RNA segments, and (2) by mediating cell-to-cell and long-distance movement, particularly with CP-RT [42,44]. Given these complexities, it was postulated that PMTV’s local and systemic movement may require major or minor CP subunits, or potentially both. Additionally, little is known about the individual roles of these components in symptom development during viral infection [28,45]. 

Here, expression of TGB1 and TGB2 individually resulted in weak to mild symptoms such as leaf curling and crumpling, while TGB3 and CP individually produced mild to moderate symptoms. Co-infiltration of CP with either TGB1 or TGB2 resulted in moderate foliar symptoms. Notably, the combination of CP and TGB3 (CP-TGB3) produced severe symptoms on leaves from 10 dpi to 14 dpi. These TGB symptoms were correlated with the relatively higher levels of RNA transcripts determined by qRT-PCR. Younger, uninoculated leaves harvested from plants infiltrated individually with CP and TGB3 showed higher expression levels compared to those infiltrated with TGB1 and TGB2. Similarly, leaves harvested from CP-TGB1 co-infiltrated plants also displayed higher gene expression levels. Additionally, symptomatic leaflets were tested using ELISA to indirectly assess if the symptoms were solely caused by PMTV genes. PMTV CP was detected in leaves infiltrated with CP-TGB1, CP-TGB2, and CP-TGB3 constructs, while PVX CP was detected in all constructs including the empty vector. In summary, plants infected with individual genes displayed mild to moderate symptoms (Table 1), whereas among the combinations, plants infiltrated with CP-TGB3 exhibited more severe symptoms (Table 1). 

## 4. Materials and Methods

### 4.1. Plant Material and Gene Amplification

*N. benthamiana* seeds were grown in Sunshine Mix LC1 (Sun Gro Horticulture, Agawam, MA, USA) under controlled conditions at 22 °C with 120 µmol m^−2^ s^−2^ under a 16 h light/8 h dark cycle. Five-week-old plants were used for inoculation. Plants were kept in the dark overnight in a growth chamber prior to inoculation. PMTV CP and TGB encoded genes were amplified from PMTV-positive tubers collected from the field. Total RNA was extracted using an RNeasy Plant Mini Kit (Qiagen, Germantown, MD, USA) following the manufacturer’s instructions. Purified RNA was then reverse transcribed to generate cDNA using a RevertAid first strand cDNA synthesis kit (ThermoFisher Scientific, Waltham, MA, USA) with an oligo (dT) primer. PCR was performed to amplify CP and TGB encoded genes using Phusion high-fidelity DNA polymerase (ThermoFisher Scientific). Positive clones for all assays were confirmed by sequencing using gene-specific primers (Table 2).

For PMTV coded gene expression, PVX-derived vector pGR106 was used [28], which was obtained from Sir David Baulcombe (Plant Department, University of Cambridge, UK) [46]. The amplified products of CP and TGB encoded genes possessed *ClaI*/*SalI* sites, which were purified and ligated with the pGR106 binary vector that was digested with the same enzymes. Recombinant clones were confirmed by PCR and DNA sequencing. All constructs were separately transformed into *Agrobacterium tumefaciens* strain GV3101 (pSoup) using electroporation and grown on LB agar plates supplemented with rifampicin 25 µg/mL and kanamycin 50 µg/mL. Selected colonies were grown in LB liquid culture with 100 µM of acetosyringone, kanamycin, and rifampicin. At 0.8 of OD_600_, bacterial cultures were harvested and resuspended in infiltration buffer (10 mM MgCl_2_, 10 mM MES, 100 µM acetosyringone) which was further incubated for 3 hrs at room temperature. Three to five leaves were infiltrated using a 1 mL needless syringe. After 7, 10, and 14 days post inoculation (dpi), young leaves were harvested and snap frozen for analysis.

### 4.2. Quantitative Reverse Transcriptase PCR

Total RNA was isolated from leaf tissue using an RNeasy Plant Mini Kit (Qiagen) following the manufacturer’s instructions. To remove any DNA contamination, RNA samples were treated with RNase-free DNase I and then reverse transcribed to synthesize cDNA. These RNA samples were also used for determining the accumulation of PVX-CP, PVX-12K, and PVX-25K, in addition to the detection of the PMTV genes. A quantitative reverse transcriptase polymerase chain reaction (qRT-PCR) of TGB genes and CP was carried out on a CFX96 Real-Time System (Bio-Rad, Hercules, CA, USA) using a SsoAdvanced Universal SYBR Green Supermix Kit (Bio-Rad). The PCR profile consisted of polymerase activation and an initial DNA denaturation cycle set at 95 °C for 3 min, followed by 44 cycles of a denaturation step at 95 °C for 15 s and an annealing/extension step at 60 °C for 20 s (according to template size and polymerase efficiency). At least two biological and two technical replicates were used. Elongation factor 1-alpha (EF1-α) and phosphatase 2A (PP2A) were used as endogenous controls for normalization as described previously [47]. Primers are listed in Table 2. The gene expression levels of all four genes relative to a reference gene were calculated using the 2^−∆∆Ct^ method [48].

### 4.3. Double Antibody Sandwich Enzyme-Linked Immunosorbent Assay (DAS-ELISA)

A DAS-ELISA assay was performed using antisera specific for PVX and PMTV (Bioreba AG, Reinach, Switzerland). Lyophilized positive controls consisted of PMTV- and PVX-infected plant tissue, while negative controls were extracts from healthy plants. The cut-off value was chosen using the formula “mean value + 3 × standard deviation + 10%” to discriminate potential positive samples from those free of PVX and PMTV. Student’s *t* test was used to determine statistical significance.

## 5. Conclusions

The phenotypic and molecular data presented here demonstrate that CP and TGB3 potentially act in coordination in inducing symptoms during PMTV infection. Identifying the role of RNA-2 and RNA-3 during asymptomatic infection and the development of spraing symptoms inside tubers remains to be investigated.

## Figures and Tables

**Table 1:** Response of *Nicotiana benthamiana* to infiltration by various PMTV constructs.

Constructs	Symptoms	qRT-PCR	ELISA
PMTV-CP	Modest symptoms—curling, crumpling, necrosis	Positive	Positive
PMTV-TGB1	Mild symptoms—curling and crumpling	Positive	Negative for CP
PMTV-TGB2	Mild symptoms—curling and crumpling	Positive	Negative for CP
PMTV-TGB3	Higher mild to modest symptoms—curling, crumpling, necrosis	Positive	Negative for CP
PMTV- CP-TGB1	Higher mild to modest symptoms—curling, crumpling, necrosis	Positive (Both)	Positive for CP
PMTV- CP-TGB2	Higher mild to modest symptoms—curling, crumpling, necrosis	Positive (Both)	Positive for CP
PMTV-CP-TGB3	Modest to severe symptoms—curling, crumpling, necrosis	Positive (Both)	Positive for CP
Vector only	Light green chlorosis area	Negative	Negative

**Table 2:** List of primers used in this study.

Primers	Sequences ^a^	
PMTV- TGB1	F = ATCGATATGGAAAGTGGATTCAACGGAAG R = GTCGACTTATTCCGGACCATACCTGTCTG	For cloning and sequencing
PMTV- TGB2	F = ATCGATATGATGGTCCGGAATAACGAAATTGG R = GTCGACTTAGCTTCCATATGACCTGCAGCAG	
PMTV- TGB3	F = ATCGATATGGATCCTCCAGTAATAATACAT R = GTCGACTTAATAACGAGCTAACATAGCACCC	
PMTV- CP	F = ATCGATATGGCTGAAAACAGAGGTGAGCG R = GTCGACCTATGCACCAGCCCAGCGTAAC	
PMTV- TGB1	F = CCGCAGGCTCAAGCGTGAGAAT R = GGGCTTACCAGTAGATTTGAATCCAC	For detection propose ^b^
PMTV- TGB2	F = TAAATTTGCTAACGGTGGCCAG R = AGCAGTCGCCTCTACATGT	
PMTV- TGB3	F = TTTCAGGAATTTCCTTATGGGAATATTCCTT R = ACGAGCTAACATAGCACCCAC	
PMTV- CP	F = AAAACAGAGGTGAGCGAAGAGCA R = AGACACCTGGCTCAACACGCTAGTGGC	

^a^ Nucleotides underlined indicate restriction sites for ClaI and SalI. ^b^ The PCR efficiencies for primer sets were as follows: 100.84% for PMTV-TGB1, 99.90% for PMTV-TGB2, 99.61% for PMTV-TGB3 and 100.24% for PMTV-CP.

## Data Availability

Data are contained within the article.

## References

[1] Beals K.A. (2019). Potatoes, Nutrition and Health. Am. J. Potato Res..

[2] Taylor R.J., Pasche J.S., Gudmestad N.C. (2008). Susceptibility of Eight Potato Cultivars to Tuber Infection by Phytophthora Erythroseptica and Pythium Ultimum and Its Relationship to Mefenoxam-mediated Control of Pink Rot and Leak. Ann. Appl. Biol..

[3] Wang H., Trusch F., Turnbull D., Aguilera-Galvez C., Breen S., Naqvi S., Jones J.D., Hein I., Tian Z., Vleeshouwers V. (2021). Evolutionarily Distinct Resistance Proteins Detect a Pathogen Effector through Its Association with Different Host Targets. New Phytol..

[4] Mora-Romero G.A., Félix-Gastélum R., Bomberger R.A., Romero-Urías C., Tanaka K. (2022). Common Potato Disease Symptoms: Ambiguity of Symptom-Based Identification of Causal Pathogens and Value of on-Site Molecular Diagnostics. J. Gen. Plant Pathol..

[5] Sandgren M., Savenkov E.I., Valkonen J.P.T. (2001). The Readthrough Region of Potato Mop-Top Virus (PMTV) Coat Protein Encoding RNA, the Second Largest RNA of PMTV Genome, Undergoes Structural Changes in Naturally Infected and Experimentally Inoculated Plants. Arch. Virol..

[6] Nielsen S.L., Nicolaisen M. (2003). Identification of Two Nucleotide Sequence Sub-Groups within Potato Mop-Top Virus. Arch. Virol..

[7] Jones R.A.C., Harrison B.D. (1969). The Behaviour of Potato Mop-top Virus in Soil, and Evidence for Its Transmission by *Spongospora subterranea* (Wallr.) Lagerh. Ann. App Biol..

[8] Arif M., Torrance L., Reavy B. (1995). Acquisition and Transmission of Potato Mop-to Furovirus by a Culture of *Spongospora subterranea* f. Sp. *Subterranea derived* from a Single Cystosorus. Ann. Appl. Biol..

[9] Arif M., Reavy B., Torrance L. (1999). Read-Through Protein Gene of Potato Mop-Top Furovirus Is Associated with Acquisition and Transmission of the Virus by *Spongospora subterranea* f. Sp. *Subterranea*. Pak. J. Bot..

[10] Pandey B., Mallik I., Gudmestad N.C. (2020). Development and Application of a Real-Time Reverse-Transcription PCR and Droplet Digital PCR Assays for the Direct Detection of Potato Mop Top Virus in Soil. Phytopathology.

[11] Valkonen J.P. (2007). Viruses: Economical Losses and Biotechnological Potential. Potato Biology and Biotechnology.

[12] Swisher Grimm K.D., Quick R.A., Cimrhakl L., Brown C., Pavek M.J. (2022). Detection of Potato Mop-Top Virus in Potato Seed Lots Entering Washington State. Am. J. Potato Res..

[13] Falloon R., Kirkwood I., Delmiglio C., Bleach C., Monk J., Clelland S. (2024). Potato Mop-Top Virus: Knowledge Review, and Evaluation of the Biosecurity Response to ‘Incursion’ of This Virus in New Zealand. N. Z. Plant Prot..

[14] Sandgren M. (1995). Potato Mop-Top Virus (PMTV): Distribution in Sweden, Development of Symptoms during Storage and Cultivar Trials in Field and Glasshouse. Potato Res..

[15] Savenkov E.I., Sandgren M., Valkonen J.P. (1999). Complete Sequence of RNA 1 and the Presence of tRNA-like Structures in All RNAs of Potato Mop-Top Virus, Genus Pomovirus. J. Gen. Virol..

[16] Santala J., Samuilova O., Hannukkala A., Latvala S., Kortemaa H., Beuch U., Kvarnheden A., Persson P., Topp K., Ørstad K. (2010). Detection, Distribution and Control of Potato Mop-top Virus, a Soil-borne Virus, in Northern Europe. Ann. Appl. Biol..

[17] Savenkov E.I., Germundsson A., Zamyatnin A.A., Sandgren M., Valkonen J.P. (2003). Potato Mop-Top Virus: The Coat Protein-Encoding RNA and the Gene for Cysteine-Rich Protein Are Dispensable for Systemic Virus Movement in Nicotiana Benthamiana. J. Gen. Virol..

[18] Samuilova O., Santala J., Valkonen J.P. (2013). Tyrosine Phosphorylation of the Triple Gene Block Protein 3 Regulates Cell-to-Cell Movement and Protein Interactions of Potato Mop-Top Virus. J. Virol..

[19] Cerovska N., Moravec T., Rosecka P., Filigarova M., Pecenkova T. (2003). Nucleotide Sequences of Coat Protein Coding Regions of Six Potato Mop-Top Virus Isolates. Acta Virol..

[20] Čeřovská N., Pečenková T., Filigarová M., Dědič P. (2007). Sequence Analysis of the Czech Potato Mop-Top Virus (PMTV) Isolate Korneta-Nemilkov. Folia Microbiol..

[21] Latvala-Kilby S., Aura J.M., Pupola N., Hannukkala A., Valkonen J.P. (2009). Detection of Potato Mop-Top Virus in Potato Tubers and Sprouts: Combinations of RNA2 and RNA3 Variants and Incidence of Symptomless Infections. Phytopathology.

[22] Budziszewska M., Wieczorek P., Nowaczyk K., Borodynko N., Pospieszny H., Obrepalska-Steplowska A. (2010). First Report of Potato Mop-Top Virus on Potato in Poland. Plant Dis..

[23] Crosslin J.M. (2011). First Report of Potato Mop-Top Virus on Potatoes in Washington State. Plant Dis..

[24] Whitworth J.L., Crosslin J.M. (2013). Detection of Potato Mop Top Virus (Furovirus) on Potato in Southeast Idaho. Plant Dis..

[25] Ramesh S.V., Raikhy G., Brown C.R., Whitworth J.L., Pappu H.R. (2014). Complete Genomic Characterization of a Potato Mop-Top Virus Isolate from the United States. Arch. Virol..

[26] Zamyatnin A.A., Solovyev A.G., Savenkov E.I., Germundsson A., Sandgren M., Valkonen J.P., Morozov S.Y. (2004). Transient Coexpression of Individual Genes Encoded by the Triple Gene Block of Potato Mop-Top Virus Reveals Requirements for TGBp1 Trafficking. Mol. Plant-Microbe Interact..

[27] Cowan G.H., Roberts A.G., Chapman S.N., Ziegler A., Savenkov E.I., Torrance L. (2012). The Potato Mop-Top Virus TGB2 Protein and Viral RNA Associate with Chloroplasts and Viral Infection Induces Inclusions in the Plastids. Front. Plant Sci..

[28] Haupt S., Cowan G.H., Ziegler A., Roberts A.G., Oparka K.J., Torrance L. (2005). Two Plant-Viral Movement Proteins Traffic in the Endocytic Recycling Pathway. Plant Cell.

[29] Tilsner J., Cowan G.H., Roberts A.G., Chapman S.N., Ziegler A., Savenkov E., Torrance L. (2010). Plasmodesmal Targeting and Intercellular Movement of Potato Mop-Top Pomovirus Is Mediated by a Membrane Anchored Tyrosine-Based Motif on the Lumenal Side of the Endoplasmic Reticulum and the C-Terminal Transmembrane Domain in the TGB3 Movement Protein. Virology.

[30] Lukhovitskaya N.I., Yelina N.E., Zamyatnin A.A., Schepetilnikov M.V., Solovyev A.G., Sandgren M., Morozov S.Y., Valkonen J.P.T., Savenkov E.I. (2005). Expression, Localization and Effects on Virulence of the Cysteine-Rich 8 kDa Protein of Potato Mop-Top Virus. J. Gen. Virol..

[31] Gil J.F., Adams I., Boonham N., Nielsen S.L., Nicolaisen M. (2016). Molecular and Biological Characterization of Potato Mop-top Virus (PMTV, Pomovirus) Isolates from the Potato-growing Regions of Colombia. Plant Pathol..

[32] Browning I., Craigie J., Darling M., Darling D., Holmes R. Studies on the Detection, Transmission to Progeny and Symptom Expression of Potato Mop Top Virus in Potato. Proceedings of the Virology Section Meeting of EAPR.

[33] Montero-Astúa M., Vasquéz V., Turechek W.W., Merz U., Rivera C. (2008). Incidence, Distribution, and Association of *Spongospora subterranea* and Potato Mop-Top Virus in Costa Rica. Plant Dis..

[34] Needleman S.B., Wunsch C.D. (1970). A General Method Applicable to the Search for Similarities in the Amino Acid Sequence of Two Proteins. J. Mol. Biol..

[35] Smith T.F., Waterman M.S. (1981). Identification of Common Molecular Subsequences. J. Mol. Biol..

[36] Takken F.L.W., Luderer R., Gabriels S.H.E.J., Westerink N., Lu R., de Wit P.J.G.M., Joosten M.H.A.J. (2000). A Functional Cloning Strategy, Based on a Binary PVX-Expression Vector, to Isolate HR-Inducing cDNAs of Plant Pathogens. Plant J..

[37] Carnegie S.F., Cameron A.M., McCreath M. (2010). Foliar Symptoms Caused by Potato Mop-Top Virus on Potato Plants during Vegetative Propagation in Scotland and Their Association with Tuber Yield, Spraing and Tuber Infection. Potato Res..

[38] Carnegie S.F., Davey T., Saddler G.S. (2010). Effect of Temperature on the Transmission of Potato Mop-top Virus from Seed Tuber and by Its Vector, *Spongospora subterranea*. Plant Pathol..

[39] Davey T., Carnegie S.F., Saddler G.S., Mitchell W.J. (2014). The Importance of the Infected Seed Tuber and Soil Inoculum in Transmitting Potato Mop-Top Virus to Potato Plants. Plant Pathol..

[40] Lucas W.J. (2006). Plant Viral Movement Proteins: Agents for Cell-to-Cell Trafficking of Viral Genomes. Virology.

[41] Lim H.-S., Bragg J.N., Ganesan U., Lawrence D.M., Yu J., Isogai M., Hammond J., Jackson A.O. (2008). Triple Gene Block Protein Interactions Involved in Movement of Barley Stripe Mosaic Virus. J. Virol..

[42] Torrance L., Lukhovitskaya N.I., Schepetilnikov M.V., Cowan G.H., Ziegler A., Savenkov E.I. (2009). Unusual Long-Distance Movement Strategies of Potato Mop-Top Virus RNAs in Nicotiana Benthamiana. Mol. Plant-Microbe Interact..

[43] Verchot-Lubicz J., Torrance L., Solovyev A.G., Morozov S.Y., Jackson A.O., Gilmer D. (2010). Varied Movement Strategies Employed by Triple Gene Block–Encoding Viruses. Mol. Plant-Microbe Interact..

[44] Torrance L., Wright K., Crutzen F., Cowan G., Lukhovitskaya N., Bragard C., Savenkov E. (2011). Unusual Features of Pomoviral RNA Movement. Front. Microbiol..

[45] Kamal H., Zafar M.M., Razzaq A., Ijaz A., Anvar Z., Topçu H., Elhindi K.M., Saeed A., Fatima U., Jiang X. (2024). Using computational modeling to design antiviral strategies and understand plant-virus interactions. Turk. J. Agric. For..

[46] Lu R., Malcuit I., Moffett P., Ruiz M.T., Peart J., Wu A.-J., Rathjen J.P., Bendahmane A., Day L., Baulcombe D.C. (2003). High Throughput Virus-Induced Gene Silencing Implicates Heat Shock Protein 90 in Plant Disease Resistance. EMBO J..

[47] Kamal H., Lynch-Holm V., Pappu H.R., Tanaka K. (2024). Starch Plays a Key Role in Sporosorus Formation by the Powdery Scab Pathogen *Spongospora subterranea*. Phytopathology.

[48] Schmittgen T.D., Livak K.J. (2008). Analyzing Real-Time PCR Data by the Comparative CT Method. Nat. Protoc..

